# Chronic obstructive pulmonary disease in East Africa: a systematic review and meta-analysis

**DOI:** 10.1093/inthealth/ihae011

**Published:** 2024-02-07

**Authors:** Guesh Mebrahtom, Abrha Hailay, Teklewoini Mariye, Teklehaimanot Gereziher Haile, Goitom Girmay, Kidane Zereabruk, Woldu Aberhe, Degena Bahrey Tadesse

**Affiliations:** Department of Adult Health Nursing, College of Health Science, School of Nursing, Aksum University, Aksum, Ethiopia; Department of Adult Health Nursing, College of Health Science, School of Nursing, Aksum University, Aksum, Ethiopia; Department of Adult Health Nursing, College of Health Science, School of Nursing, Aksum University, Aksum, Ethiopia; Department of Maternity and Neonatal Nursing, College of Health Science, School of Nursing, Aksum University, Aksum, Ethiopia; Department of Clinical Midwifery, College of Health Science, Aksum University, Aksum, Ethiopia; Department of Adult Health Nursing, College of Health Science, School of Nursing, Aksum University, Aksum, Ethiopia; Department of Adult Health Nursing, College of Health Science, School of Nursing, Aksum University, Aksum, Ethiopia; Department of Adult Health Nursing, College of Health Science, School of Nursing, Aksum University, Aksum, Ethiopia

**Keywords:** chronic obstructive pulmonary disease, East Africa, meta-analysis

## Abstract

Chronic obstructive pulmonary disease (COPD) is a common lung disease that causes restricted airflow and breathing problems. Globally, COPD is the third leading cause of death and low- and middle-income countries account for the majority of these deaths. There is limited information on COPD's prevalence in East Africa. Thus the purpose of this systematic review and meta-analysis is to estimate the pooled prevalence of COPD in East Africa.A computerized systematic search using multiple databases was performed in search of relevant English articles from the inception of the databases to August 2023. All the authors independently extracted the data. R and RStudio software were used for statistical analysis. Forest plots and tables were used to represent the data. The statistical heterogeneity was evaluated using I^2^ statistics. There was heterogeneity between the included articles. Therefore, a meta-analysis of random effects models was used to estimate the overall pooled prevalence of COPD in East Africa. A funnel plot test was used to examine possible publication bias.The database search produced 512 papers. After checking for inclusion and exclusion criteria, 43 full-text observational studies with 68 553 total participants were found suitable for the review. The overall pooled prevalence of COPD in East Africa was 13.322%. The subgroup analysis found the COPD pooled prevalence in the different countries was 18.994%, 7%, 15.745%, 9.032%, 15.026% and 11.266% in Ethiopia, Uganda, Tanzania, Malawi, Sudan, and Kenya, respectively. Additionally, the subgroup analysis of COPD by study setting among community-based studies was 12.132% and 13.575% for hospital-based studies.According to the study's findings, approximately one of every seven individuals in East Africa has COPD, indicating a notably high prevalence of the disease. Thus governments and other stakeholders working on non-communicable disease control should place an emphasis on preventive measures to minimize the burden of COPD.

## Introduction

Chronic obstructive pulmonary disease (COPD) is a common and progressive chronic respiratory condition resulting in gradual deterioration and worsening of symptoms and mainly characterized by obstruction of airflow in the lungs.^1^ Shortness of breath, coughing up mucus and persistent respiratory symptoms are the main symptoms of this preventable disease that is characterized by airflow limitation due to abnormalities in the airways and/or alveoli, usually brought on by significant exposure to noxious particles or gases.^2^ Although preventable, once established, it cannot be cured, but effective self-management strategies can lessen the burden of disease and improve quality of life.^3^

According to the Global Burden of Disease (GBD) Study, in 2017, all-age prevalent cases of chronic respiratory disease were 545 million, of which about 50% were due to chronic obstructive pulmonary disease (COPD), which is responsible for 3.2 million deaths^4^ and was the fourth leading cause of mortality in the USA. According to the World Health Organization (WHO), in 2011 almost 90% of COPD deaths occurred in low- and middle-income countries (LMICs), where effective strategies for prevention and control are not always implemented or accessible. It is usually associated with inflammatory reactions, comorbidities and risk factors including genetics, smoking, infections, malnutrition, aging, occupational exposures, indoor and outdoor air pollutants, asthma and low socio-economic status.^1,4,5^ Cigarette smoking is the leading cause of COPD. Most people who have COPD smoke or used to smoke. Long-term exposure to other lung irritants, such as air pollution, chemical fumes or dust, also may contribute to COPD. Emphysema and chronic bronchitis are the two most common conditions that contribute to COPD.^5^ The main contributor of increased morbidity and death in those with COPD is coronavirus disease 2019 (COVID-19).^6,7^ Patients with COPD have an increased risk of neuronal injury, lung infection, pneumothorax, poor gas exchange, osteoporosis, polycythaemia, hypoxia or associated comorbidities, cognitive impairment and cardiovascular diseases.^8^ COPD results in productivity losses due to compromised quality of life among working-age patients.^2^ Systematic reviews on the prevalence of COPD in Africa are inconsistent, ranging from 1–17% by Njoku et al.^9^ to 9.4–22.1% by Adeloye et al.^10^ However, none of the reviews on the prevalence of COPD in Africa gave data by region or country. Also, reports of the prevalence of COPD from cross-sectional studies in East Africa are inconsistent, ranging from 1.05% by Alıcı and Genç^11^ to 54.6% by Anbesse et al.^12^ in Somalia and Ethiopia, respectively.

Many factors contribute to this huge difference, including study quality, study design, ethnic differences, geographic location and different diagnostic methods, as well as the emerging of COVID-19 in those countries. Even though some studies have been published on the prevalence COPD in East Africa,^13–17^ the general prevalence remains unknown. Hence, having a pooled prevalence will help to overcome these discrepancies and provide a common understanding and enhance awareness of the prevention and management of COPD. Therefore, the purpose of this systematic review and meta-analysis is to estimate the pooled prevalence of COPD in East Africa.

## Methods

### Study protocol and systematic review registration

This review was based on the Preferred Reporting Items for Systematic Reviews and Meta-Analyses (PRISMA) guideline^18^ (Supplementary Material 1). This review has not been registered in the International Prospective Register of Systematic Reviews (PROSPERO) database.

### Study setting

This review was conducted in nine countries in the East African Region: Ethiopia, Kenya, Madagascar, Malawi, Rwanda, Sudan, Tanzania, Uganda and Somalia.^19^

### Study design

We conducted a systematic review and meta-analysis that included all studies that report the prevalence of COPD in East African nations. The studies that were included were all observational. Six of the studies were cohort studies, one was a case–control study and the remaining studies were cross-sectional.

### Search strategy and information sources

Electronic databases that were searched include PubMed, Google Scholar, Web of Science, Cochrane Library, Africa Wide Information, Africa Index Medicus, Africa Journal Online and the World Health Organization (WHO) Afro Library from inception to August 2023. The existence of prior systematic reviews or protocols on the topic of interest was investigated by searching the Cochrane Database of Systematic Reviews, Joanna Briggs Institute Database of Systematic Reviews and Implementation Reports (JBI-DSRIR), National Institute for Health Research Centre for Reviews and Dissemination Database, Health Technology Assessment (HTA) database, Campbell Collaboration and Evidence for Policy and Practice Information and Co-ordinating Centre. The literature search technique was developed using the headings of the medical subject headings (Met); a Boolean (AND/OR) operator was used. The combination of key terms including ‘COPD’, ‘chronic obstructive pulmonary disease’, chronic obstructive lung disease’ ‘asthma complication’, ‘chronic airflow obstructions’, ‘airway obstructions’, ‘chronic bronchitis’, ‘emphysema’, ‘chronic cigarette smoking’, ‘nation name’, ‘systematic review’ and protocols were used. The search from the above databases confirmed that there was no systematic review and/or protocol on the topic of interest.

### Data extraction and quality assessment

With the use of a pretested data extraction format created in an Excel 2016 spreadsheet (Microsoft, Redmond, WA, USA) and a structured method of data collecting, all the authors independently extracted the data. Two reviewers (GM and AH) independently examined the titles, abstracts of all retrieved citations and full-text search results to categorize possibly qualifying articles. Title, first author's name, year of data collection, year of publication, study design, study setting (community or hospital-based), COPD screening or diagnostic approach used in the studies, number of cases (individuals with COPD), total/sample size, prevalence/incidence rate of COPD, response rate and study country were extracted from the studies. Furthermore, appropriate sampling techniques, consistent data collection techniques, recorded quality control methods and a representative sample size were all considered as indicators of the study quality.

### Criteria for considering studies for the review

#### Inclusion criteria

The inclusion criteria were the study design (all published observational studies), patient age ≥15 y, only peer-reviewed articles, both hospital-based and community-based studies, English-language articles, method of diagnosis (studies that used spirometry for diagnosis in the majority of the studies and studies that reported the prevalence of COPD using patient records without stating their diagnostic criteria) and the prevalence of COPD.

#### Exclusion criteria

The following studies were excluded from consideration: case reports, case series studies, studies lacking the pertinent information required to calculate the prevalence of COPD and studies conducted in patients <15 y of age.

### Quality assessment and risk of bias in individual studies

Methodological quality was assessed using the Newcastle–Ottawa scale^20^ (Supplementary Material 3). The primary purpose of this scale is to evaluate the risk of bias through a star allocation system that allows up to 10 stars in three categories: study group selection (4 or 5 stars), group comparability (2 stars) and exposure or outcome determination (3 stars). No validation study provides a cut-off score for rating low-quality studies; a priori, we arbitrarily established that 0–3, 4–6 and 7–10 stars would be considered at high, moderate and low risk of bias, respectively. No study was excluded because of methodological bias. Therefore, all of the studies were included in this review. For each included study, the precision or margin of error (C) was estimated, taking into account the sample size (SS) and the observed prevalence (p) of COPD using the formula: SS=z2∗p∗(1−p)d2, where z represented the z-value fixed at 1.96 across studies (corresponding to a 95% confidence interval [CI]). The desirable margin of error is ≥5%.

### Data management

According to the inclusion and exclusion criteria, a tool was created to direct the screening and selection process. The tool was piloted and modified prior to beginning data extraction. In order to remove duplicate studies, the search results were uploaded to Endnote software (Clarivate, London, UK).

### Data analysis and presentation of results

Relevant data from every study extracted in the Excel spreadsheet was exported to R version 4.3.1 and RStudio version 2023.06.1+524 software (R Foundation for Statistical Computing, Vienna, Austria), both used as data analysis software. Forest plots were drawn in order to calculate the pooled estimate prevalence of COPD and the degree of statistical heterogeneity between studies. The pooled estimate was computed using the ‘meta prop’ command. Statistical heterogeneity was assessed using the standard χ^2^ test (Cochrane's Q test) and quantified by calculating the I^2^ statistic (with values of 25%, 50% and 75% being representative of low, medium and high heterogeneity, respectively).^21^ There was heterogeneity between the included studies in this study. Consequently, we used a random effects meta-analysis to estimate the overall pooled prevalence of COPD in East Africa; this might be due to variations in the study location, differences in the sample size and differences in the objective of the studies. Funnel plot test methods were used to assess possible publication bias. Subgroup analysis was conducted for the country where the study was conducted and the study setting, which was community-based or hospital-based.

### Study selection and data collection process

Studies that were carried out in East Africa and reported the prevalence/incidence of COPD were selected for the meta-analysis. Two authors independently reviewed the titles and abstracts of articles that were taken from the reviewed articles to check if they met the inclusion criteria after the findings had been gathered. Disagreements were resolved by discussion following agreement with the help of a third independent reviewer (DBT). When there was missing information, the corresponding author of the study was contacted to request the missing information. A maximum of three e-mails were sent to the corresponding author of the retrieved studies to request additional information before excluding the study. For studies that occurred in multiple publications, we used the one that was the most recent, comprehensive and with the greatest sample size. For surveys that appeared in a single article with several surveys done at various times, we treated each survey as a separate study.

### Data items

Data extraction included the following: title, first author, data collection year, year published, country, study setting or area, study design, number of cases, total or sample size, response rate, prevalence or incidence rate of COPD and diagnostic or screening approach to COPD.

### Outcomes and prioritization

The primary outcome was the prevalence of COPD among countries in East Africa.

### Data synthesis

A meta-analysis was performed to estimate the pooled prevalence of COPD in East Africa. Results were presented using forest plots. Subgroup analysis was summarized by the country where the study was conducted and by the study setting, which was community-based or hospital-based.

Because there was heterogeneity among the studies, a random effects model^22^ was used to determine the pooled prevalence of COPD in East Africa. Heterogeneity was explored using Cochrane's Q and quantified by I^2^ statistics.^23^ Results were reported as proportions with corresponding 95% CIs. The results of this review were reported based on the PRISMA guidelines.^18^

## Results

### Screening flow

**Figure 1.**
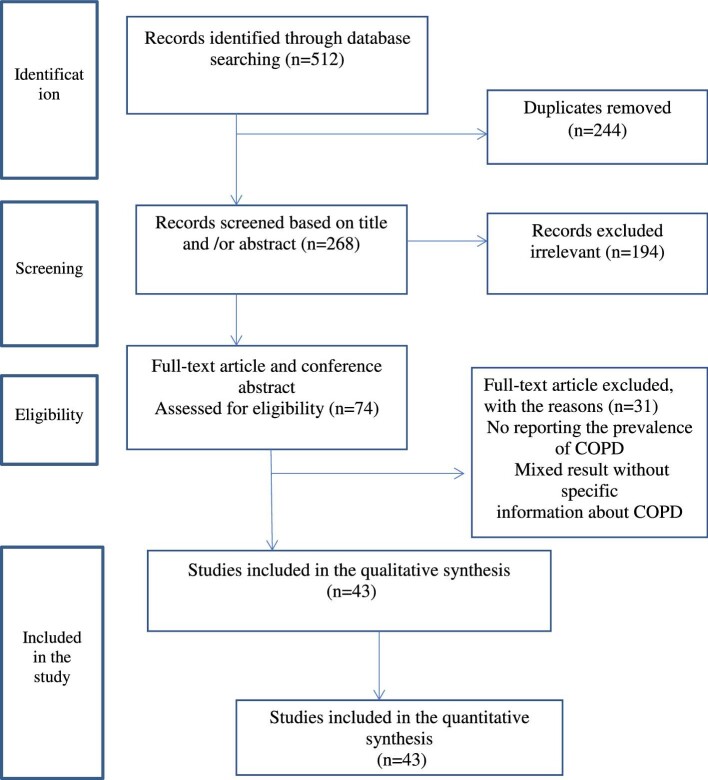
Flow chart showing the selection of articles for systemic review and meta-analysis of COPD in East Africa, 2023.

Figure 1 is a flow diagram outlining the process of identification and selection of the included studies. The included databases and number of included studies were PubMed (n=161), Google Scholar (n=216), Cochran Library (n=42), Africa-Wide Information (n=2), WHO Afro library (n=8), African Journal online (n=37), Web of Science (n=34) and African Index Medicus (n=12). Based on the predefined criteria and quality assessment, 244 duplicates were identified and removed. Subsequently, we screened 268 titles and abstracts and excluded 194 irrelevant articles. A total of 74 articles and conference abstracts were assessed for eligibility criteria and 31 of those did not report the prevalence of COPD. Finally, 43 full-text articles with 68 553 total participants were included in this systematic review and meta-analysis.

### Study characteristics

More than one-third (15 [34.88%]) of the studies were conducted in Ethiopia, followed by 8 (18.6%) from Uganda, 5 (11.62%) from Tanzania, 4 (9.3%) from Sudan, 4 (9.3%) from Malawi, 4 (9.3%) from Kenya, 1 (2.32%) from Rwanda, 1 (2.32%) from Madagascar and 1 (2.32%) from Somalia. Six studies were cohort studies and one was a case–control study; the rest were cross-sectional. Of these studies, eight were community-based and the rest were hospital-based. A total of 39 (90.7%) of the studies employed spirometry to diagnose COPD, compared with just 4 (9.3%) studies that did not specify their diagnostic approach and instead used patient charts. The quality of each primary study assessed using the Newcastle–Ottawa Scale shows no significant risk. Therefore, all the included studies considered in this systematic review and meta-analysis had a low risk of bias (Table 1).

**Table 1.** Study characteristics of the included articles for the final systematic review and meta-analysis of COPD in East Africa, 2023

Authors	Data collection year	Year published	Country	Study setting	Study design	Participants with COPD, n	Sample size, n	Prevalence rate, %	Response rate, %	Diagnostic criteria	Quality assessment based on NOS
Woldeamanuel et al.^13^	2019	2019	Ethiopia	Hospital	Cs	131	734	17.8	94.2	Spirometry	9
Parekh et al.^23^	2014	2015	Ethiopia	Hospital	Cs	14	249	6	82	Spirometry	8
Binegdie et al.^24^	2017	2020	Ethiopia	Hospital	Cs	41	99	4.4	100	Spirometry	9
Binegdie et al^24^	2014	2015	Ethiopia	Hospital	Cs	2637	46 422	5.7	99.7	Spirometry	9
Alıcı and Genç^11^	2019	2020	Ethiopia	Hospital	Co	71	130	54.6	86.7	Spirometry	7
Maoz et al.^26^	2007	2008	Ethiopia	Hospital	CC	43	223	19.3	100	Spirometry	8
Kaso et al.^27^	2021	2022	Ethiopia	Hospital	Cs	55	398	13.8	100	From chart	9
Kaso et al.^28^	2021	2022	Ethiopia	Hospital	Co	48	422	11.4	100	From chart	9
Boka et al.^29^	2022	2023	Ethiopia	Hospital	Co	58	508	11.4	100	From chart	9
Kaso et al.^30^	2021	2022	Ethiopia	Hospital	Cs	37	493	7.5	100	From chart	9
Mega et al.^31^	2019	2020	Ethiopia	Hospital	Cs	71	130	54.6	80	Spirometry	7
Agedew et al.^32^	2020	2021	Ethiopia	Community	Cs	65	615	10.6	96.4	Spirometry	9
Binegdie et al.^33^	2020	2021	Ethiopia	Hospital	Cs	26	170	15.3	100	Spirometry	8
Araya et al.^34^	2021	2022	Ethiopia	Hospital	Co	9	440	2	100	Spirometry	9
Garcia-Vidal et al.^14^	2008	2009	Kenya	Hospital	Cs	56	146	38.4	100	Spirometry	7
Ombajo et al.^35^	2020	2021	Kenya	Hospital	Co	21	787	3	100	Spirometry	9
Navuluri et al.^36^	2019	2023	Kenya	Hospital	Cs	12	305	3.93	100	Spirometry	9
Binegdie et al.^33^	2020	2021	Kenya	Hospital	Cs	3	209	1.4	100	Spirometry	8
Fullerton et al.^37^	2010	2011	Malawi	Community	Cs	45	331	13.6	88.5	Spirometry	9
Njoroge et al.^38^	2019	2021	Malawi	Community	Co	134	1082	12.4	83	Spirometry	9
Allyn et al.^39^	2013	2016	Madagascar	Hospital	Cs	9	62	14.5	100	Spirometry	7
Banda et al.^40^	2015	2017	Malawi	Hospital	Cs	25	503	4.97	100	Spirometry	9
Sichali et al.^41^	2015	2019	Malawi	community	Cs	35	608	5.8	100	Spirometry	9
Tadesse et al.^42^	2015	2016	Ethiopia	Hospital	Cs	20	90	18	100	Spirometry	8
Musafiri et al.^43^	2010	2011	Rwanda	Hospital	Cs	82	1824	4.5	95	Spirometry	9
Muthana et al.^44^	2008	2008	Sudan	Hospital	Cs	27	59	44	100	Spirometry	7
Ahmed^17^	2016	2018	Sudan	Hospital	Cs	54	1308	4.1	100	Spirometry	9
Osman^45^	2013	2014	Sudan	Hospital	Cs	12	136	8.8	100	Spirometry	9
Binegdie et al.^33^	2020	2021	Sudan	Hospital	Cs	12	140	140	100	Spirometry	8
Onesmo et al.^46^	2022	2023	Tanzania	Community	Cs	33	103	32.04	100	Spirometry	8
Magitta et al.^47^	2017	2019	Tanzania	Community	Cs	83	1021	8.13	100	Spirometry	8
Magitta et al.^16^	2016	2018	Tanzania	Hospital	Cs	152	869	17.5	100	Spirometry	9
Mwaiselage et al.^48^	2002	2005	Tanzania	Hospital	Cs	27	222	12.2	100	Spirometry	8
Knudsen^49^	2008	2010	Tanzania	Hospital	Cs	41	326	12.6	100	Spirometry	9
North et al.^50^	2015	2019	Uganda	Hospital	Cs	17	843	2	98	Spirometry	9
Siddharthan et al.^51^	2016	2019	Uganda	Community	Cs	65	1502	4.3	100	Spirometry	9
van Gemert^15^	2012	2017	Uganda	Community	Cs	95	588	16.2	94.8	Spirometry	9
North et al.^52^	2015	2017	Uganda	Hospital	Cs	9	239	4	89	Spirometry	8
Kayongo et al.^53^	2017	2020	Uganda	Hospital	Cs	45	722	6.22	100	Spirometry	9
Ddungu et al.^54^	2017	2021	Uganda	Hospital	Cs	9	288	3.1	100	Spirometry	9
Gilbert et al.^55^	2018	2022	Uganda	Hospital	Cs	17	265	6.4	92	Spirometry	9
Kayongo et al.^56^	2021	2023	Uganda	Hospital	Cs	200	1378	14.51	100	Spirometry	9
Alıcı and Genç^11^	2020	2021	Somalia	Hospital	Cs	15	1428	1.05	100	Spirometry	9

CC: case–control; Cs: cross-sectional; Co: cohort; NOS: Newcastle–Ottawa Scale.

### The pooled prevalence of COPD in East Africa

**Figure 2.**
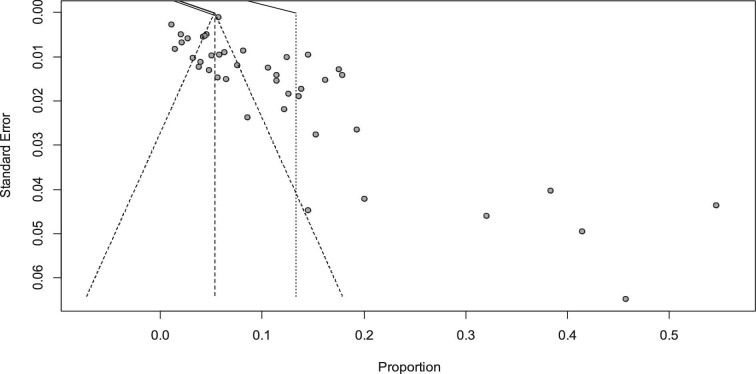
A funnel plot shows an asymmetric distribution, which is evidence of publication bias across studies.

In the analysis of 43 studies according to the random effects model, the pooled prevalence of COPD in East Africa was 13.322% (95% CI 9.456 to 17.187) (Supplementary Material 4) and the inverse variance (I^2^) was 97%, indicating heterogeneity in the reported prevalence of COPD among the included studies. This heterogeneity might be due to differences in the population's geographic location and ethnicity, differences in socio-economic and educational status, variations in industrialization of the countries, differences in the prevalence of smoking and environmental conditions of the countries and individual differences in the screening and diagnosis of COPD. A leave-one-out sensitivity analysis was carried out to see whether the findings of a single study had a significant impact on the pooled prevalence of COPD in East Africa. However, all the results of this sensitivity analysis were within the 95% CI limits of the pooled prevalence (9.456 to 17.187), indicating that no significant study may have had an impact on the observed pooled prevalence of COPD (Supplementary Material 5). A funnel plot was used to test the publication bias and showed an asymmetrical distribution (Figure 2), which indicates evidence of publication bias in the included studies. Hence there are unpublished articles that could modify the pooled prevalence of COPD.

### Subgroup analysis of COPD prevalence by study countries in East Africa

**Figure 3.**
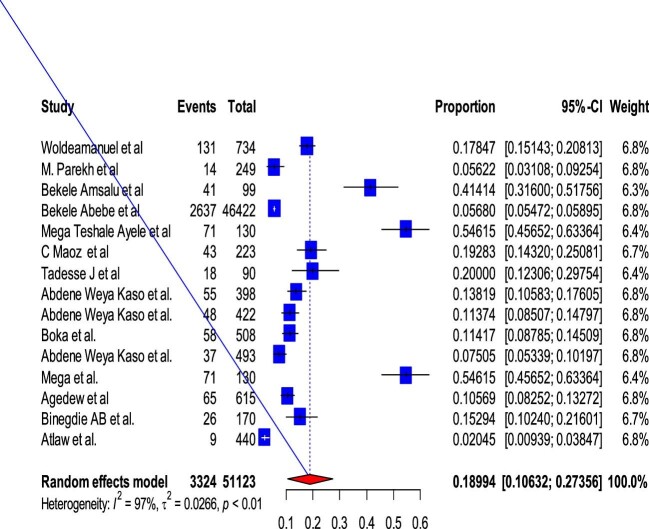
A forest plot shows the pooled prevalence of COPD in Ethiopia from 15 observational studies (18.994% [95% CI 10.632 to 27.356]).

**Figure 4.**
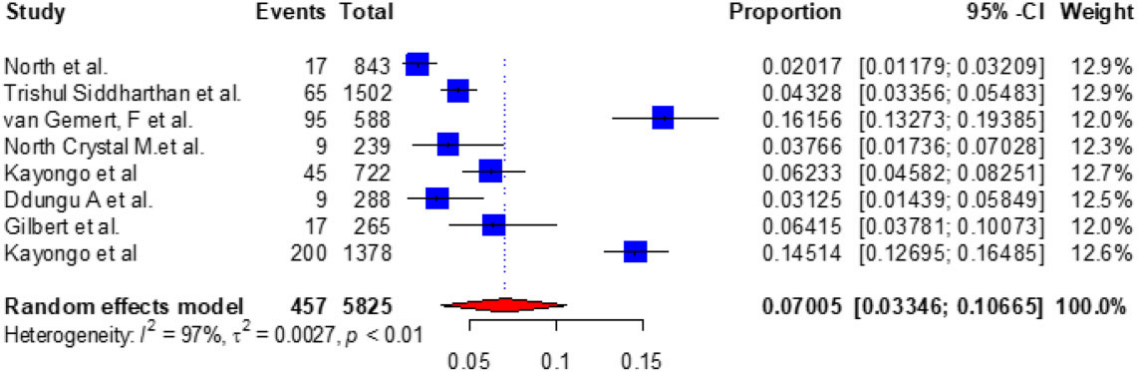
A forest plot shows the pooled prevalence of COPD in Uganda from eight observational studies (7.005% [95% CI 3.346 to 10.665]).

**Figure 5.**
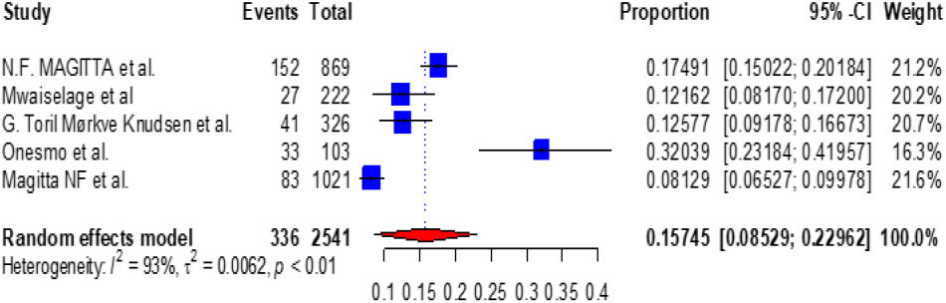
A forest plot shows the pooled prevalence of COPD in Tanzania from five observational studies (15.745% [95% CI 8.529 to 22.692]).

**Figure 6.**
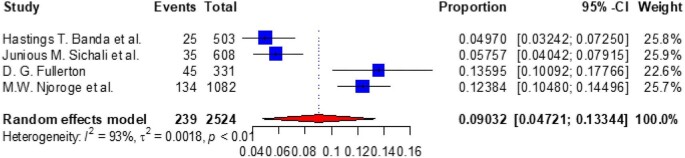
A forest plot shows the pooled prevalence of COPD in Malawi from four observational studies (9.032% [95% CI 4.721 to 13.344]).

**Figure 7.**
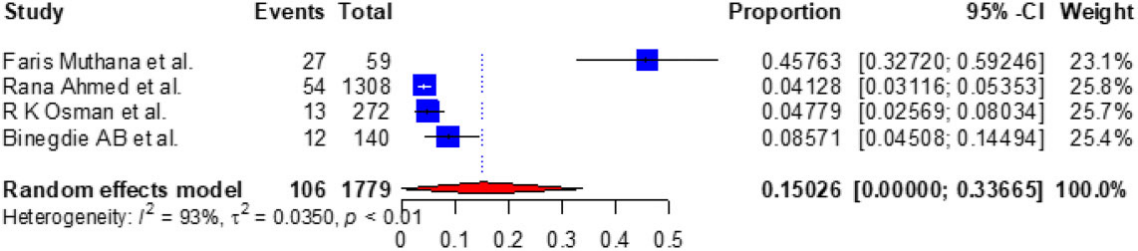
A forest plot shows the pooled prevalence of COPD in Sudan from four observational studies (15.026% [95% CI 0 to 33.665]).

**Figure 8.**
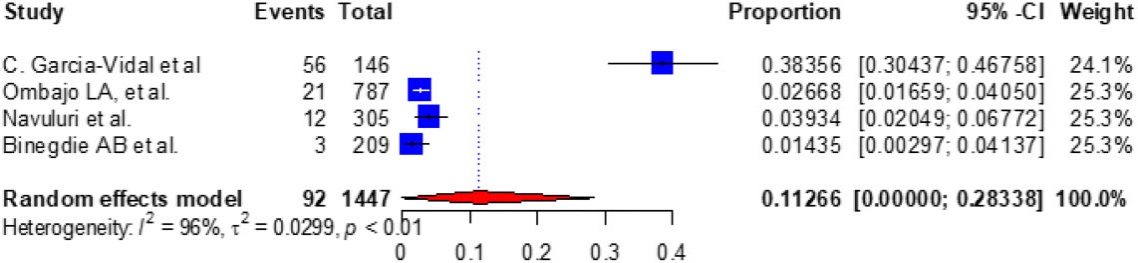
A forest plot shows the pooled prevalence of COPD in Kenya from four observational studies (11.266% [95% CI 0 to 28.338]).

Based on the subgroup analysis of COPD by the study country, the pooled point estimate prevalence of COPD in Ethiopia was 18.994% (95% CI 10.632 to 27.356) (Figure 3), in Uganda it was 7% (95% CI 3.346 to 10.665) (Figure 4), in Tanzania it was 15.745% (95% CI 8.529 to 22.962) (Figure 5), in Malawi it was 9.032% (95% CI 4.721 to 13.344) (Figure 6), in Sudan it was 15.026% (95% CI 0 to 33.665) (Figure 7) and in Kenya it was 11.266% (95% CI 0 to 28.338) (Figure 8).

### Subgroup analysis of the prevalence of COPD in East Africa by study setting

**Figure 9.**
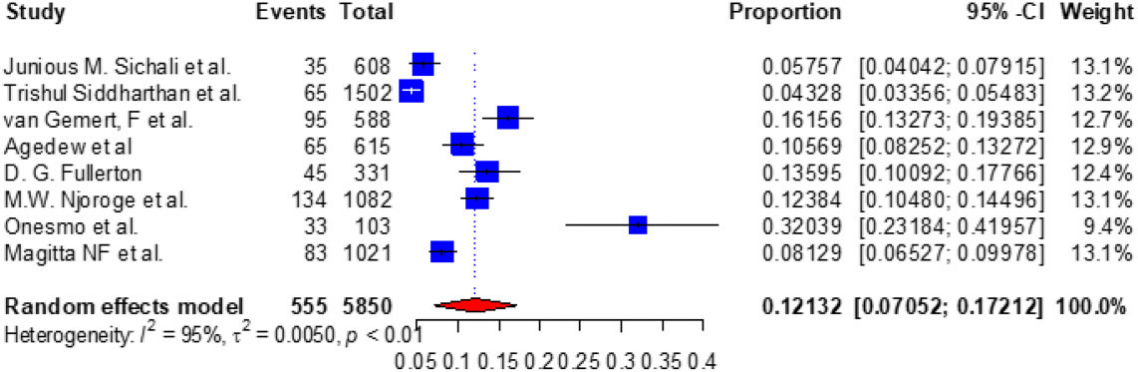
A forest plot shows the pooled prevalence of COPD in East Africa among eight community-based observational studies (12.132% [95% CI 7.052 to 17.212]).

**Figure 10.**
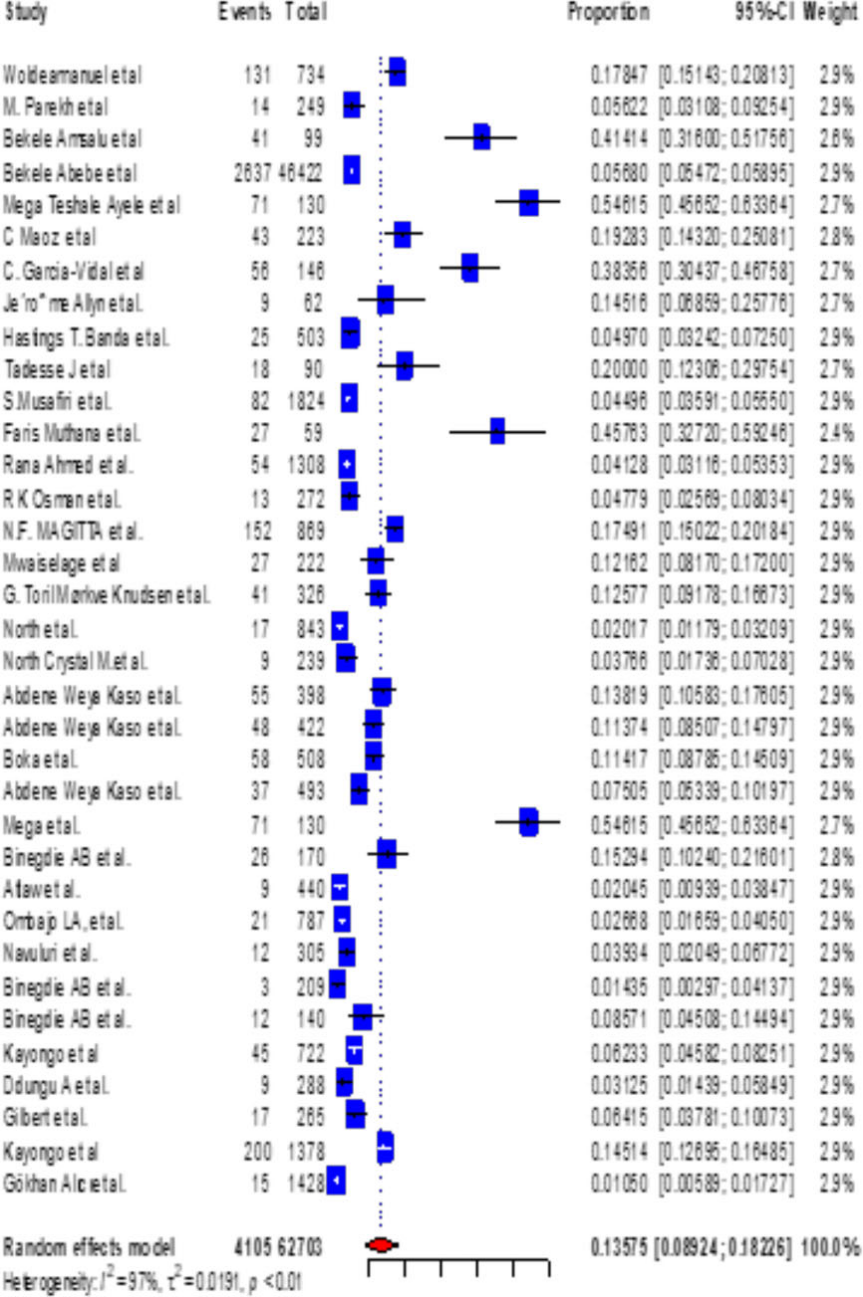
A forest plot shows the pooled prevalence of COPD in East Africa among 35 hospital-based observational studies (13.575% [95% CI 8.924 to 18.226]).

The subgroup analysis of COPD by study setting was 12.132% (95% CI 7.052 to 17.212) for community-based studies (Figure 9) and 13.575% (95% CI 8.924–18.226) for hospital-based studies (Figure 10).

## Discussion

COPD is one of the leading causes of morbidity and mortality in industrialized and developing countries, with most of the deaths coming from LMICs.^57–59^ The primary cause of COPD is exposure to tobacco smoke. Other factors associated with COPD, especially in Africa, are environmental air pollution; exposure to combustion products of biomass fuel; occupational dusts, vapours and fumes; previous pulmonary tuberculosis (TB), human immunodeficiency virus (HIV) infection, poor diet, malnutrition (including effects in utero), low socio-economic status and childhood respiratory infections, and COPD is likely to increase in the coming years due to higher smoking prevalence and aging populations in many countrie.^60–62^ In addition, urbanization and westernization of the continent, in particular East Africa, have increased the prevalence of non-communicable diseases like COPD.^63^

A total of 43 studies were included in the final analysis, providing an overall pooled prevalence of COPD in East Africa of 13.322% (95% CI 9.456 to 17.187). Individually, there was variation in the prevalence of COPD in the studies included in our review, ranging from 1.05% in Somalia^11^ to 54.6% in Ethiopia.^12^ This variation could be explained by differences in the objectives and study designs, number of participants, ethnicity of the population, geographic location, level of industrialization and prevalence of smoking, lifestyle and behavioural characteristics and socio-economic status of the population.^64–66^

The overall pooled prevalence of COPD in this study (13.322%) was consistent with a study reported on the global prevalence of COPD in 2010 that had a pooled prevalence of 11.7%^67^ and a systematic review from Africa published in 2015 that reported the difference in the median prevalence of COPD in persons ≥40 y of age based on spirometry and non-spirometry data (13.4% and 4.0%, respectively).^10^ A possible explanation for this could be the shared experiences of uncontrolled urbanization, increasing life expectancy or increasing age of the African population, exposure to both indoor and outdoor air pollution, socio-economic similarities among the African population and the fact that LMICs account for the majority of the world's COPD prevalence.^60,61,68^

Similarly, the results of this systematic review and meta-analysis were comparable with reports of systematic reviews conducted in China, where the prevalence was 13.6%,^69^ in Brazil, where it was 17%,^70^ and in the global prevalence, where it was 12.6% in 2019.^71^ This could be explained by the sociodemographic similarities between the two populations, since the majority of the prevalence of COPD in China was from rural areas, just as the East African populations live in rural areas, and the majority of COPD cases in Brazil were found in the centre-west region of the country, where agriculture accounts for the majority of the economy.^72^

The results of this systematic review and meta-analysis were higher compared with the global prevalence of 7.6% reported in 2006^73^ and the reports of systematic reviews conducted in China, where the prevalence was 10.0%,^74^ in sub-Saharan Africa, where it was 8%,^75^ and in Latin America, where it was 8.9%.^76^ This could be explained by differences in objectives, the number of participants, age of the participants, ethnicity of the population, geographic location, prevalence of smoking, lifestyle and behavioural characteristics of the population, environmental conditions and socio-economic and educational status of the population.^66,77^

Adeloye et al.,^10^ Finney et al.^78^ and Mehrotra et al.^79^ conducted systematic reviews in Africa that highlight the significant burden of COPD on the continent and the need for additional research to improve understanding regarding the disease's prevalence, mortality and risk factors. Additionally, there is a lack of essential resources, such as spirometers, which are important for COPD diagnosis and care. However, they were unable to estimate the prevalence of COPD by country or region in East Africa. The small number of studies and different objectives included in these reports could explain this.

To look at the heterogeneity of the studies included in this review, we conducted subgroup analysis by country and we found pooled prevalences of COPD of 18.994% (95% CI 10.632 to 27.356), 7.005% (95% CI 3.346 to 10.665), 15.745% (95% CI 8.529 to 22.962), 11.266% (95% CI 0.0 to 28.338), 9.032% (95% CI 4.721 to 13.334) and 15.026% (95% CI 0.0 to 33.665) in Ethiopia, Uganda, Tanzania, Kenya, Malawi and Sudan, respectively. The discrepancy in COPD prevalence in East African countries may be due to differences in the study populations, study settings (urban vs rural) or study locations (community vs hospital); participant age, prior history of TB, exposure to biomass smoke, presence of comorbidities like asthma, bronchitis and HIV and the prevalence of smoking; variations in the population's geographic location, ethnographic background and socio-economic status; variations in the industrialization of the countries; the impacts of other health determinants and the fact that risk factors cannot be generalized.^10,61,75,78,80^

Additionally, we did a subgroup analysis of the prevalence of COPD in East African nations for community-based and hospital-based studies. As a result, we discovered that the prevalence of COPD in communities was 12.132% (95% CI 8.924 to 18.226) and in hospitals it was 13.575% (95% CI 8.924 to 18.226), which is comparable to the combined total of COPD prevalence in East Africa.

Our meta-analysis results have implications for clinical practice since they may enhance COPD patient care and preventive measures as well as act as important health and safety indicators. This pooled estimate for COPD provides up-to-date data to promote preventative strategies and the requirement for including resources that assist people in conquering challenges to reduce the occurrence of this chronic disease. The importance of this study, particularly the pronounced discrepancy among countries, reflects actions that require developing standards for prevention strategies and establishing guidelines for clinical practice. In this systematic review, the most common contributing factors to COPD were poor housing and ventilation, indoor and outdoor air pollution, cigarette smoking, exposure to toxic or biomass fumes and underlying diseases such as chronic bronchitis, asthma and prior pulmonary TB.

The findings of our study on the prevalence of COPD in this region could assist with prevention strategies such as avoiding tobacco exposure (both active and passive measures), biomass/toxic fumes, quitting smoking, eliminating or reducing workplace exposures, and early treatment and management of underlying diseases like chronic bronchitis, asthma, pulmonary TB, etc. To lessen the effects of air pollution and COPD, health initiatives should be introduced, such as congestion charging, high-occupancy vehicle lanes and encouraging walking or cycling.^81^

This is the first systematic review and meta-analysis of the pooled prevalence of COPD in East African countries. However, the findings of this study have certain limitations. Among these, subgroup analysis for studies between countries was difficult to carry out due to statistical constraints and the limited number of studies, which is why we conducted subgroup analysis of Ethiopia, Uganda, Tanzania, Kenya, Malawi and Sudan. This makes our review subject to a high degree of heterogeneity between studies, as we did not include all countries in the study. However, the model of random effects was used to achieve the pooled results, thus minimizing this heterogeneity among studies. A limitation is also that only English-language studies were searched. In addition, methodological and clinician variations in assessments of COPD among studies could also affect meta-analysis results, with extensive clinical heterogeneity across studies. This study was also limited in evaluating the pooled factors causing COPD.

## Conclusions

The findings from this study show approximately one of seven individuals in East Africa suffers by COPD. This finding indicates that COPD is significantly common in East Africa. The countries ministries of health, health policymakers, the WHO, clinicians and other healthcare providers should focus on strengthening COPD preventive measures and establish condition-based and country-specific preventive measures to reduce the burden of COPD. The authors of this study recommend further research on the factors causing COPD in the region.

## Supplementary Material

ihae011_Supplemental_Files

## Data Availability

The data analysed in the current meta-analysis are available from the corresponding author upon reasonable request.
