# Supplementary material for: Chronic obstructive pulmonary disease in East Africa: a systematic review and meta-analysis
Source: Int Health. 2024 Feb 7;16(5):499–511. doi: 10.1093/inthealth/ihae011 (PMC11375591; doi:10.1093/inthealth/ihae011)
Supplement: ihae011_Supplemental_Files [file ihae011_supplemental_files.zip › Supplementary material 2.docx]

**Supplementary material 2**

**Search Strategy and Information Sources**

A search strategy was implemented using electronic databases (PubMed, Google Scholar, Web of Science, Cochrane library, Africa Wide Information, and Africa Index Medicus, Africa journal online, World Health Organization (WHO) Afro library) from inception to August 2023. The literature search technique was developed using the headings of the medical subject headings (Met), BOOLEAN (AND/OR) operator was used. The combination of key terms including “COPD”, “Chronic Obstructive Pulmonary Disease”, Chronic Obstructive lung Disease” “Asthma Complication”, "chronic airflow obstructions", "airway obstructions", “Chronic Bronchitis”, “Emphysema”, “Chronic Cigarette smoking”, “Nation Name”, “systematic review” and protocols were used. The presence of precursor systematic review and/or protocol on the topic of interest was checked via searching different databases for the systematic review. The databases searched include the Cochrane database of a systematic review, Joanna Briggs Institute database of a systematic review and implementation reports (JBI-DSRIR), the national health center review and dissemination database, health technology assessment-HTA, the Campbell collaboration library and evidence for policy and practice information (EPPI-center). The search from the above databases confirmed that there was no systematic review and /or protocol on the topic of interest.

| Serial number | Databases | Number of articles found | Number of articles included | Number of Excluded articles | Reason for exclusion |
| --- | --- | --- | --- | --- | --- |
| 1 | PubMed | n=161 | n=21 | n=140 | Duplicates(n=110) and Irrelevant papers (n=30) |
| 2 | Google Scholar | n=216 | n= 18 | n=198 | Duplicates (n= 123)  and Irrelevant papers (n=75) |
| 3 | Cochran Library | n=42 | n=0 | n=42 | Duplicates(n=3) and Irrelevant papers (n=39) |
| 4 | Africa wide information | n=2 | n=0 | n=2 | Irrelevant papers |
| 5 | World Health Organization (WHO) afro library | n=8 | n=0 | n=8 | Irrelevant papers |
| 6 | African journal online | 37 | n=4 | n=33 | Duplicates(n=8) and Irrelevant papers (n= 25) |
| 7 | Web of Science | n=34 | N=0 | n=34 | Irrelevant papers |
| 8 | African Index Medicus (AID) | 12 | n=0 | n=12 | Irrelevant papers |

The included databases and number of included studies thereof were PubMed (161), Google Scholar (216), Cochran Library (42), Africa wide information (2), the World Health Organization (WHO) afro library (8), African Journal online (37), Web of Science (34), and African Index Medicus (12). Of those 244 duplicates were identified and removed. Subsequently, we screened 268 titles and abstracts and excluded 225 irrelevant papers. Then, based on the pre-defined criteria and quality assessment, 43 full-text observational study articles with 68553 total participants were included in this systematic review and meta-analysis.
