# Supplementary material for: Chronic obstructive pulmonary disease in East Africa: a systematic review and meta-analysis
Source: Int Health. 2024 Feb 7;16(5):499–511. doi: 10.1093/inthealth/ihae011 (PMC11375591; doi:10.1093/inthealth/ihae011)
Supplement: ihae011_Supplemental_Files [file ihae011_supplemental_files.zip › Supplementary Material 3.docx]

**Supplementary Material 3. Newcastle-Ottawa quality assessment scale**

**S1 Text**

**NEWCASTLE - OTTAWA QUALITY ASSESSMENT SCALE**

**(Adapted for cross sectional studies)**

**Selection:** (Maximum 5 stars)

1) Representativeness of the sample:

1. Truly representative of the average in the target population. * (all subjects or random sampling)
2. Somewhat representative of the average in the target population. * (non-random sampling)
3. Selected group of users.
4. No description of the sampling strategy.

2) Sample size:

1. Justified and satisfactory. *
2. Not justified.

3) Non-respondents:

1. Comparability between respondents and non-respondents’ characteristics is established, and the response rate is satisfactory. *
2. The response rate is unsatisfactory, or the comparability between respondents and non-respondents is unsatisfactory.
3. No description of the response rate or the characteristics of the responders and the non-responders.
4. Ascertainment of the exposure (risk factor): a) Validated measurement tool. **

b) Non-validated measurement tool, but the tool is available or described* c) No description of the measurement tool.

**Comparability:** (Maximum 2 stars)

1. The subjects in different outcome groups are comparable, based on the study design or analysis. Confounding factors are controlled.

a) The study controls for the most important factor (select one). * b) The study control for any additional factor. *

**Outcome:** (Maximum 3 stars)

1) Assessment of the outcome:

1. Independent blind assessment. **
2. Record linkage. **
3. Self-report. *
4. No description.

2) Statistical test:

1. The statistical test used to analyze the data is clearly described and appropriate, and the measurement of the association is presented, including confidence intervals and the probability level (p value). *
2. The statistical test is not appropriate, not described or incomplete.

This scale has been adapted from the Newcastle-Ottawa Quality Assessment Scale for cohort studies to perform a quality assessment of cross-sectional studies for the systematic review, “Are Healthcare Workers’ Intentions to Vaccinate Related to their Knowledge, Beliefs and Attitudes? A Systematic Review”.

We have not selected one factor that is the most important for comparability, because the variables are not the same in each study. Thus, the principal factor should be identified for each study.

In our scale, we have specifically assigned one star for self-reported outcomes, because our study measures the intention to vaccinate. Two stars are given to the studies that assess the outcome with independent blind observers or with vaccination records, because these methods measure the practice of vaccination, which is the result of true intention.

CODING MANUAL FOR CASE-CONTROL STUDIES

***SELECTION***

1. **Is the Case Definition Adequate?**
2. Requires some independent validation (e.g., >1 person/record/time/process to extract information, or reference to primary record source such as x-rays or medical/hospital records)
3. Record linkage (e.g., ICD codes in database) or self-report with no reference to primary record
4. No description
5. **Representativeness of the Cases**
6. All eligible cases with outcome of interest over a defined period of time, all cases in a defined catchment area, all cases in a defined hospital or clinic, group of hospitals, health maintenance organization, or an appropriate sample of those cases (e.g., random sample)
7. Not satisfying requirements in part (a), or not stated.
8. **Selection of Controls**

This item assesses whether the control series used in the study is derived from the same population as the cases and essentially would have been cases had the outcome been present.

1. Community controls (i.e. same community as cases and would be cases if had outcome)
2. Hospital controls, within same community as cases (i.e. not another city) but derived from a hospitalized population
3. No description
4. **Definition of Controls**
5. If cases are first occurrence of outcome, then it must explicitly state that controls have no history of this outcome. If cases have new (not necessarily first) occurrence of outcome, then controls with previous occurrences of outcome of interest should not be excluded.
6. No mention of history of outcome

# COMPARABILITY

1. **Comparability of Cases and Controls on the Basis of the Design or Analysis**

## A maximum of 2 stars can be allotted in this category

Either cases and controls must be matched in the design and/or confounders must be adjusted for in the analysis. Statements of no differences between groups or that differences were not statistically significant are not sufficient for establishing comparability. Note: If the odds ratio for the exposure of interest is adjusted for the confounders listed, then the groups will be considered to be comparable on each variable used in the adjustment.

There may be multiple ratings for this item for different categories of exposure (e.g. ever vs. never, current vs. previous or never)

Age = , Other controlled factors =

# EXPOSURE

1. **Ascertainment of Exposure**

Allocation of stars as per rating sheet

1. **Non-Response Rate**

Allocation of stars as per rating sheet

**CODING MANUAL FOR COHORT STUDIES**

# SELECTION

1. **Representativeness of the Exposed Cohort**

Item is assessing the representativeness of exposed individuals in the community, not the representativeness of the sample of women from some general population. For example, subjects derived from groups likely to contain middle class, better educated, health-oriented women are likely to be representative of postmenopausal estrogen users while they are not representative of all women (e.g., members of a health maintenance organization (HMO) will be a representative sample of estrogen users. While the HMO may have an under-representation of ethnic groups, the poor, and poorly educated, these excluded groups are not the predominant users of estrogen).

Allocation of stars as per rating sheet

1. **Selection of the Non-Exposed Cohort**

Allocation of stars as per rating sheet

1. **Ascertainment of Exposure**

Allocation of stars as per rating sheet

1. **Demonstration That Outcome of Interest Was Not Present at Start of Study**

In the case of mortality studies, outcome of interest is still the presence of a disease/ incident, rather than death. That is to say that a statement of no history of disease or incident earns a star.

***COMPARABILITY***

1. **Comparability of Cohorts on the Basis of the Design or Analysis**

A maximum of 2 stars can be allotted in this category

Either exposed and non-exposed individuals must be matched in the design and/or confounders must be adjusted for in the analysis. Statements of no differences between groups or that differences were not statistically significant are not sufficient for establishing comparability. Note: If the relative risk for the exposure of interest is adjusted for the confounders listed, then the groups will be considered to be comparable on each variable used in the adjustment.

There may be multiple ratings for this item for different categories of exposure (e.g. ever vs. never, current vs. previous or never)

Age = , Other controlled factors =

***OUTCOME***

1. **Assessment of Outcome**

For some outcomes (e.g. fractured hip), reference to the medical record is sufficient to satisfy the requirement for confirmation of the fracture. This would not be adequate for vertebral fracture outcomes where reference to x-rays would be required.

1. Independent or blind assessment stated in the paper, or confirmation of the outcome by reference to secure records (x-rays, medical records, etc.)
2. Record linkage (e.g. identified through ICD codes on database records)
3. Self-report (i.e. no reference to original medical records or x-rays to confirm the outcome)
4. No description.
5. **Was Follow-Up Long Enough for Outcomes to Occur**

An acceptable length of time should be decided before quality assessment begins (e.g. 5 yrs. for exposure to breast implants)

1. **Adequacy of Follow Up of Cohorts**

This item assesses the follow-up of the exposed and non-exposed cohorts to ensure that losses are not related to either the exposure or the outcome.

Allocation of stars as per rating sheet
