# Supplementary material for: Chronic obstructive pulmonary disease in East Africa: a systematic review and meta-analysis
Source: Int Health. 2024 Feb 7;16(5):499–511. doi: 10.1093/inthealth/ihae011 (PMC11375591; doi:10.1093/inthealth/ihae011)

Supplementary Material 4: A forest plot shows the pooled prevalence of COPD in East Africa from forty-three observational studies which was 13.322% (95% CI: 9.456 - 17.187).

Study	Events	Total	Proportion	95%−CI	Weight
Woldeamanuel et al	131	734	0.17847	[0.15143; 0.20813]	2.4%
M. Parekh et al	14	249	0.05622	[0.03108; 0.09254]	2.4%
Bekele Amsalu et al	41	99	0.41414	[0.31600; 0.51756]	2.1%
Bekele Abebe et al	2637	46422	0.05680	[0.05472; 0.05895]	2.4%
Mega Teshale Ayele et al	71	130	0.54615	[0.45652; 0.63364]	2.1%
C Maoz et al	43	223	0.19283	[0.14320; 0.25081]	2.3%
C. Garcia−Vidal et al	56	146	0.38356	[0.30437; 0.46758]	2.2%
Je´roˆ me Allyn et al.	9	62	0.14516	[0.06859; 0.25776]	2.1%
Hastings T. Banda et al.	25	503	0.04970	[0.03242; 0.07250]	2.4%
Junious M. Sichali et al.	35	608	0.05757	[0.04042; 0.07915]	2.4%
Tadesse J et al	18	90	0.20000	[0.12306; 0.29754]	2.2%
S.Musafiri et al.	82	1824	0.04496	[0.03591; 0.05550]	2.4%
Faris Muthana et al.	27	59	0.45763	[0.32720; 0.59246]	1.9%
Rana Ahmed et al.	54	1308	0.04128	[0.03116; 0.05353]	2.4%
R K Osman et al.	13	272	0.04779	[0.02569; 0.08034]	2.4%
N.F. MAGITTA et al.	152	869	0.17491	[0.15022; 0.20184]	2.4%
Mwaiselage et al	27	222	0.12162	[0.08170; 0.17200]	2.3%
G. Toril Mørkve Knudsen et al.	41	326	0.12577	[0.09178; 0.16673]	2.4%
North et al.	17	843	0.02017	[0.01179; 0.03209]	2.4%
Trishul Siddharthan et al.	65	1502	0.04328	[0.03356; 0.05483]	2.4%
van Gemert, F et al.	95	588	0.16156	[0.13273; 0.19385]	2.4%
North Crystal M.et al.	9	239	0.03766	[0.01736; 0.07028]	2.4%
Abdene Weya Kaso et al.	55	398	0.13819	[0.10583; 0.17605]	2.4%
Abdene Weya Kaso et al.	48	422	0.11374	[0.08507; 0.14797]	2.4%
Boka et al.	58	508	0.11417	[0.08785; 0.14509]	2.4%
Abdene Weya Kaso et al.	37	493	0.07505	[0.05339; 0.10197]	2.4%
Mega et al.	71	130	0.54615	[0.45652; 0.63364]	2.1%
Agedew et al	65	615	0.10569	[0.08252; 0.13272]	2.4%
Binegdie AB et al.	26	170	0.15294	[0.10240; 0.21601]	2.3%
Atlaw et al.	9	440	0.02045	[0.00939; 0.03847]	2.4%
Ombajo LA, et al.	21	787	0.02668	[0.01659; 0.04050]	2.4%
Navuluri et al.	12	305	0.03934	[0.02049; 0.06772]	2.4%
D. G. Fullerton	45	331	0.13595	[0.10092; 0.17766]	2.4%
M.W. Njoroge et al.	134	1082	0.12384	[0.10480; 0.14496]	2.4%
Binegdie AB et al.	3	209	0.01435	[0.00297; 0.04137]	2.4%
Binegdie AB et al.	12	140	0.08571	[0.04508; 0.14494]	2.3%
Onesmo et al.	33	103	0.32039	[0.23184; 0.41957]	2.1%
Magitta NF et al.	83	1021	0.08129	[0.06527; 0.09978]	2.4%
Kayongo et al	45	722	0.06233	[0.04582; 0.08251]	2.4%
Ddungu A et al.	9	288	0.03125	[0.01439; 0.05849]	2.4%
Gilbert et al.	17	265	0.06415	[0.03781; 0.10073]	2.4%
Kayongo et al	200	1378	0.14514	[0.12695; 0.16485]	2.4%
Gökhan Alici et al.	15	1428	0.01050	[0.00589; 0.01727]	2.4%
**Random effects model**	**4660**	**68553**	**0.13322**	**[0.09456; 0.17187]**	**100.0%**

Heterogeneity: I^2 = 97%, τ^2 = 0.0162, p < 0.01

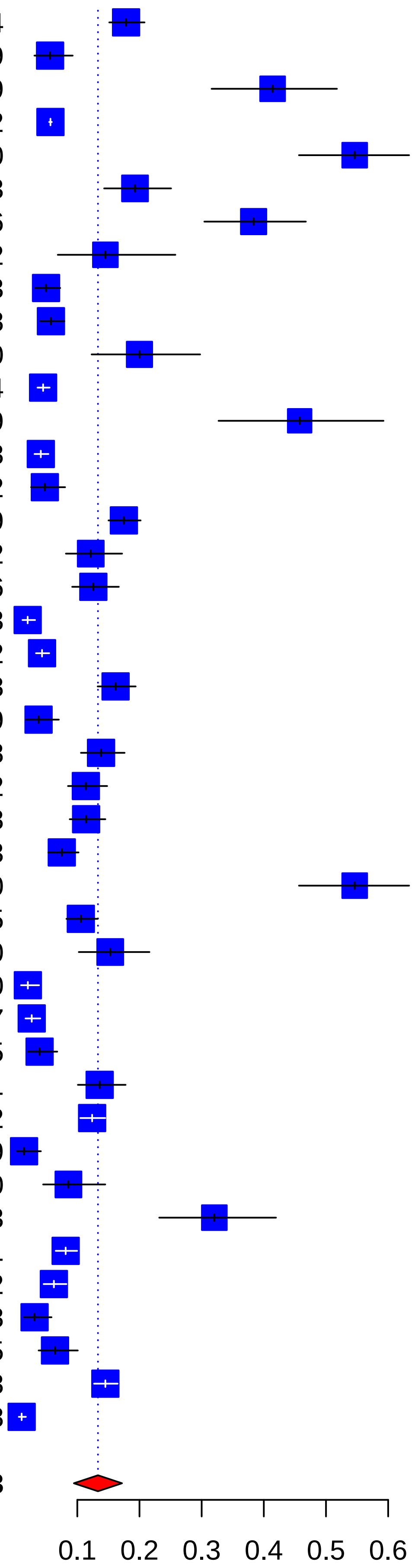

Supplement: ihae011_Supplemental_Files [file ihae011_supplemental_files.zip › Supplementary Material 4.pdf]
