# Supplementary material for: Chronic obstructive pulmonary disease in East Africa: a systematic review and meta-analysis
Source: Int Health. 2024 Feb 7;16(5):499–511. doi: 10.1093/inthealth/ihae011 (PMC11375591; doi:10.1093/inthealth/ihae011)
Supplement: ihae011_Supplemental_Files [file ihae011_supplemental_files.zip › Supplementary Material 5.pdf]

Supplementary Material 5: A forest plot for the sensitivity analysis of the studies that were included in the systematic review and meta-analysis of the pooled prevalence of COPD in East Africa, which shows that no study has significantly affected the pooled prevalence of COPD.

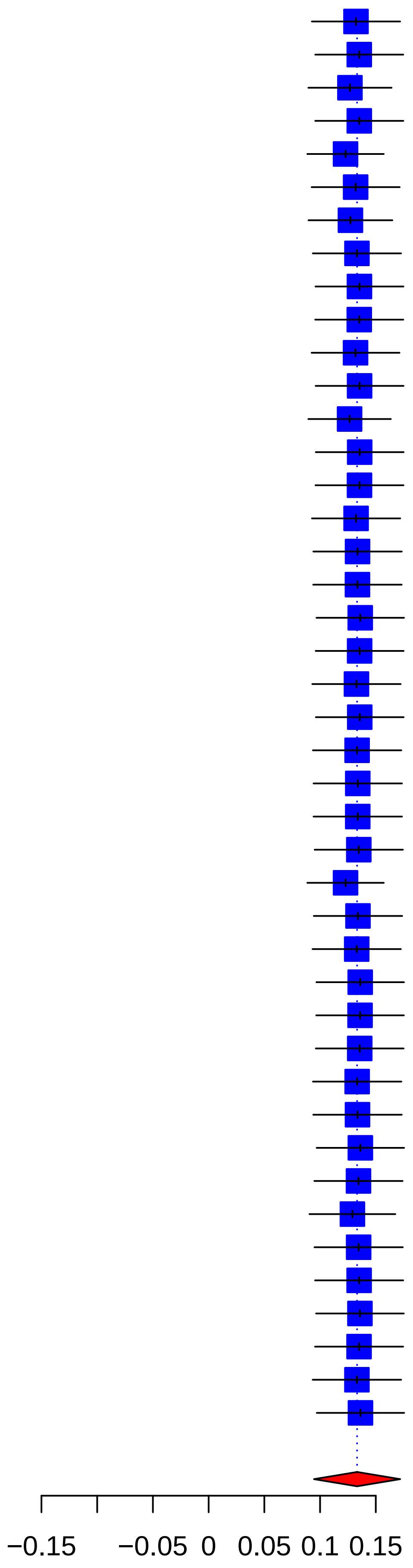

Study	Proportion	95%−CI	P−value	Tau2	Tau	I2
Omitting Woldeamanuel et al	0.13224	[0.09264; 0.17183]	.	0.0166	0.1288	97%
Omitting M. Parekh et al	0.13518	[0.09568; 0.17468]	.	0.0165	0.1285	97%
Omitting Bekele Amsalu et al	0.12680	[0.08948; 0.16413]	.	0.0147	0.1214	97%
Omitting Bekele Abebe et al	0.13519	[0.09568; 0.17470]	.	0.0165	0.1285	97%
Omitting Mega Teshale Ayele et al	0.12286	[0.08858; 0.15714]	.	0.0124	0.1112	97%
Omitting C Maoz et al	0.13192	[0.09240; 0.17143]	.	0.0165	0.1286	97%
Omitting C. Garcia−Vidal et al	0.12726	[0.08962; 0.16489]	.	0.0150	0.1224	97%
Omitting Je´roˆ me Allyn et al.	0.13308	[0.09351; 0.17265]	.	0.0166	0.1289	97%
Omitting Hastings T. Banda et al.	0.13535	[0.09588; 0.17482]	.	0.0165	0.1284	97%
Omitting Junious M. Sichali et al.	0.13517	[0.09565; 0.17468]	.	0.0165	0.1286	97%
Omitting Tadesse J et al	0.13183	[0.09239; 0.17127]	.	0.0165	0.1285	97%
Omitting S.Musafiri et al.	0.13547	[0.09602; 0.17491]	.	0.0165	0.1283	97%
Omitting Faris Muthana et al.	0.12648	[0.08944; 0.16351]	.	0.0145	0.1206	97%
Omitting Rana Ahmed et al.	0.13555	[0.09613; 0.17497]	.	0.0164	0.1283	97%
Omitting R K Osman et al.	0.13538	[0.09592; 0.17484]	.	0.0165	0.1284	97%
Omitting N.F. MAGITTA et al.	0.13233	[0.09272; 0.17193]	.	0.0166	0.1289	97%
Omitting Mwaiselage et al	0.13363	[0.09397; 0.17329]	.	0.0167	0.1291	97%
Omitting G. Toril Mørkve Knudsen et al.	0.13354	[0.09387; 0.17321]	.	0.0167	0.1291	97%
Omitting North et al.	0.13603	[0.09675; 0.17531]	.	0.0163	0.1278	97%
Omitting Trishul Siddharthan et al.	0.13550	[0.09607; 0.17494]	.	0.0165	0.1283	97%
Omitting van Gemert, F et al.	0.13266	[0.09302; 0.17230]	.	0.0166	0.1290	97%
Omitting North Crystal M.et al.	0.13561	[0.09622; 0.17501]	.	0.0164	0.1282	97%
Omitting Abdene Weya Kaso et al.	0.13324	[0.09357; 0.17291]	.	0.0167	0.1291	97%
Omitting Abdene Weya Kaso et al.	0.13383	[0.09416; 0.17350]	.	0.0167	0.1291	97%
Omitting Boka et al.	0.13382	[0.09415; 0.17349]	.	0.0167	0.1291	97%
Omitting Abdene Weya Kaso et al.	0.13475	[0.09517; 0.17434]	.	0.0166	0.1288	97%
Omitting Mega et al.	0.12286	[0.08858; 0.15714]	.	0.0124	0.1112	97%
Omitting Agedew et al	0.13403	[0.09436; 0.17369]	.	0.0167	0.1291	97%
Omitting Binegdie AB et al.	0.13289	[0.09326; 0.17251]	.	0.0166	0.1290	97%
Omitting Atlaw et al.	0.13602	[0.09674; 0.17530]	.	0.0163	0.1278	97%
Omitting Ombajo LA, et al.	0.13588	[0.09656; 0.17521]	.	0.0164	0.1279	97%
Omitting Navuluri et al.	0.13558	[0.09617; 0.17499]	.	0.0164	0.1282	97%
Omitting D. G. Fullerton	0.13329	[0.09362; 0.17296]	.	0.0167	0.1291	97%
Omitting M.W. Njoroge et al.	0.13359	[0.09390; 0.17327]	.	0.0167	0.1291	97%
Omitting Binegdie AB et al.	0.13615	[0.09692; 0.17539]	.	0.0163	0.1276	97%
Omitting Binegdie AB et al.	0.13447	[0.09487; 0.17407]	.	0.0166	0.1289	97%
Omitting Onesmo et al.	0.12902	[0.09048; 0.16756]	.	0.0157	0.1255	97%
Omitting Magitta NF et al.	0.13461	[0.09500; 0.17422]	.	0.0166	0.1289	97%
Omitting Kayongo et al	0.13506	[0.09552; 0.17459]	.	0.0165	0.1286	97%
Omitting Ddungu A et al.	0.13577	[0.09641; 0.17512]	.	0.0164	0.1280	97%
Omitting Gilbert et al.	0.13500	[0.09546; 0.17453]	.	0.0166	0.1287	97%
Omitting Kayongo et al	0.13307	[0.09339; 0.17274]	.	0.0167	0.1291	97%
Omitting Gökhan Alici et al.	0.13625	[0.09705; 0.17545]	.	0.0163	0.1275	96%
**Random effects model**	**0.13322**	**[0.09456; 0.17187]**	**.**	**0.0162**	**0.1273**	**97%**
